# Exploring the Homeopathic Therapeutic Potential of *Acorus calamus* : A Multi-center Open-Label Clinical Verification Study

**DOI:** 10.1055/a-2625-0202

**Published:** 2025-09-08

**Authors:** Suhana Panaparambil Azis, Charu Sehgal, Partha Sarathi Chakraborty, Prasanta Kumar Pradhan, Punam Kumari, Kaushal Kumar Savera, Amit Srivastava, Ratan Chandra Shil, Kumar Keshav Avinash, Sunil S. Ramteke, Amulya Ratna Sahoo, Tejaswini Patole Kamble, Uttam Singh, Mahesh Sah, Ranjit Sonny, Liyi Karso, Vibha Kumari, Divya Verma, Rakesh Kumar Rana, Kusum Lata, Beenu Saini, Satarupa Sadhukhan, Renu Verma, Ann Maria Rose, Manila Kumari, Meera Sharma, Gauri Saxena, Subhash Kaushik

**Affiliations:** 1 Department of Clinical Verification, Central Council for Research in Homeopathy Headquarters, Delhi, India; 2 Department of Clinical Verification, Dr. Anjali Chatterjee Regional Research Institute for Homeopathy, Kolkata, West Bengal, India; 3 Department of Clinical Verification, Regional Research Institute for Homeopathy, Puri, Odisha, India; 4 Department of Clinical Verification, Dr. D.P. Rastogi Central Research Institute of Homeopathy, Noida, Uttar Pradesh, India; 5 Department of Clinical Verification, National Homeopathy Research Institute in Mental Health, Kottayam, Kerala, India; 6 Department of Clinical Verification, Central Research Institute (Homeopathy), Lucknow, Uttar Pradesh, India; 7 Department of Clinical Verification, Regional Research Institute for Homeopathy, Agartala, Tripura, India; 8 Department of Clinical Verification, Clinical Verification Unit (Homeopathy), Patna, Bihar, India; 9 Department of Clinical Verification, Regional Research Institute for Homeopathy, Guwahati, Assam, India; 10 Department of Clinical Verification, Regional Research Institute for Homeopathy, Navi Mumbai, Mumbai, Maharashtra, India; 11 Department of Clinical Verification, Central Research Institute (Homeopathy), Jaipur, Rajasthan, India; 12 Department of Clinical Verification, Regional Research Institute for Homeopathy, Siliguri, West Bengal, India; 13 Statistical Section, Central Council for Research in Ayurvedic Sciences Headquarters, Delhi, India; 14 Department of Botany, University of Lucknow, Lucknow, Uttar Pradesh, India

**Keywords:** *Acorus calamus*, homeopathy, clinical verification, MYMOP-2, ORIDL

## Abstract

**Background:**

Clinical verification in homeopathy is a systematic process that aims to validate the therapeutic efficacy of homeopathic medicines. The Central Council for Research in Homoeopathy (CCRH) has been conducting clinical verification studies in lesser known, fragmentarily proved and rare medicines such as *Acorus calamus* through its Clinical Verification Program, thereby expanding the therapeutic utility of these medicines.

**Objective:**

The primary objective of the study was to assess the change in intensity of presenting symptoms of the participants after administration of the medicine *Acorus calamus* by measuring change in Measure Yourself Medical Outcome Profile-2 (MYMOP-2) profile score. The study also aimed to evaluate overall impact on participants' daily living using the Outcome in Relation to Impact on Daily Living (ORIDL) scale, as well as to identify any new clinical symptoms that were not elicited during the drug proving.

**Methods:**

A multi-center open-label study was conducted at the outpatient departments of the 14 hospitals/clinics of the CCRH located in different states of India. Participants having a minimum of three symptoms corresponding to the proving symptom of *Acorus calamus* were included in the study.

**Results:**

Improvement in the symptomatology of 321 participants who completed a minimum of two follow-ups suggested the effectiveness of the medicine in treating various clinical conditions, including headache, constipation, respiratory diseases such as rhinitis, acute nasopharyngitis, sinusitis and cough, musculoskeletal disorders such as myalgia, arthralgia and cervical spondylosis, and skin conditions such as eczema. *Acorus calamus* showed substantial therapeutic potential, with significant improvement ( *p* < 0.0001) in MYMOP-2 and in ORIDL scores. Symptoms that were not previously identified during the drug proving included sneezing and difficulty in breathing.

**Conclusion:**

The study successfully verified the symptomatology of *Acorus calamus* by ascertaining improvement in symptoms after its administration in individuals with various health conditions. Future research can focus on randomized controlled trials to quantify the efficacy of *Acorus calamus* for the indicated clinical conditions.

## Introduction

*Acorus calamus* Linn. *(* AC), commonly referred to as ‘Sweet flag’ or Calamus in English, *Bach* in Hindi and *Vacha* in Sanskrit, is a perennial aquatic plant from the Acoraceae family, native to Europe, Asia, North America, India and Sri Lanka. 1 2 3 For centuries, this striking plant has been used in traditional medicine, especially in Ayurvedic, Chinese and Western practices, owing to its potential therapeutic properties. 4 5 It is known to contain several bioactive compounds, chiefly phenylpropanoids (asarone and eugenol) and sesquiterpenoids, 5 which contribute to its diverse pharmacological effects including anti-inflammatory, 6 bronchodilatory, 7 anti-spasmodic, 8 anti-diabetic, 9 antidepressant, 5 cardioprotective, 10 hypolipidemic, 10 sedative 10 and antimicrobial 10 properties.

Despite its vast and well documented medicinal properties, AC remains surprisingly unexplored and overlooked as a valuable homeopathic remedy. In the homeopathic system of medicine, the earliest documented mention of this medicine can be found in the German Homoeopathic Pharmacopoeia. 11 Subsequently, the Central Council for Research in Homoeopathy (CCRH) undertook a comprehensive standardization of the medicine 12 13 and developed its high performance thin-layer chromatography fingerprint profile. 14 This was followed by toxicity testing of the mother tincture, which showed that doses of 0.2 mL/100 gram body weight (g.b.w.) in mice and 0.5 mL/100 g.b.w. in rats caused no adverse symptoms. 13 14 Building on this safety profile, the CCRH initiated the homeopathic drug pathogenetic (proving) trial of the medicine in healthy participants to explore its therapeutic utility. 15

In one or more participants from the different study sites during the drug pathogenetic trial, AC in various potencies produced a range of symptoms including headache, coryza, throat pain, cough, dyspnoea, pain in abdomen, constipation and musculoskeletal complaints. 15 Following the successful completion of the pathogenetic trial, CCRH systematically analyzed the generated data to design a comprehensive clinical verification study focusing on validating the proving symptoms of AC.

Clinical verification in homeopathy is a systematic process that aims to validate the therapeutic efficacy of homeopathic medicines. It involves a rigorous, methodical and confirmatory examination of symptoms previously documented in homeopathic literature as well as drug proving symptoms, thereby validating the drug pathogenesis and expanding its therapeutic applications. 16

For over four decades, CCRH has been conducting clinical verification studies through the flagship Clinical Verification Program, thereby contributing to evidence-based practices in homeopathic new medicine development. These multi-center, open-label, studies enrol participants from the CCRH's vast network of clinics/hospitals located across India, following a standardized protocol to generate reliable evidence about the effectiveness of these medicines, ultimately improving treatment options for patients.

The current paper elucidates the findings from the clinical verification study on AC. The primary objective of the study was to assess the change in symptoms of the participants after administration of the medicine AC by measuring the Measure Yourself Medical Outcome Profile-2 (MYMOP-2) 17 profile scores. Additionally, the study aimed to evaluate the overall impact on daily living by comparing changes in scores on the Outcome in Relation to Impact on Daily Living (ORIDL) 18 scale after administration of the medicine, and also to identify any new clinical symptoms that were neither observed during the proving nor found in source literature but showed significant change in participants after receiving the treatment.

## Methods

### Study Design and Setting

The study was designed as a multi-center open-label single-arm study conducted at the outpatient departments (OPDs) of the 14 hospitals/clinics of the CCRH: namely the National Homoeopathy Research Institute in Mental Health, Kottayam (NHRIMH); Dr. D.P. Rastogi Central Research Institute for Homoeopathy, Noida; Central Research Institute for Homoeopathy, Lucknow; Dr. Anjali Chatterjee Regional Research Institute for Homoeopathy, Kolkata; Regional Research Institute (H), Gudivada; Regional Research Institute (H), Mumbai; Regional Research Institute (H), Shimla; Regional Research Institute (H), Puri; Regional Research Institute (H), Imphal; Regional Research Institute (H), Guwahati; Regional Research Institute (H), Agartala; Clinical Research Unit (H), Port Blair; Clinical Verification Unit (H), Patna; and Drug Proving Research Unit for Homoeopathy, Bhubaneswar. The study sites with widespread geographic locations facilitated representation from diverse regions across the country, ensuring a broad and inclusive sample. The findings from the study are reported according to the STROBE (Strengthening the Reporting of Observational Studies in Epidemiology) guidelines. 19

### Ethical Approval

Before commencing the study, the necessary ethical clearance was obtained from the Institutional Ethics Committee of the CCRH, New Delhi (reference number: 1-3/2017- 18/CCRH/Tech./21st EC/1391; August 8, 2017).

### Participants

The participants were enrolled during the period October 2018 to May 2022. Due to the imposed lockdown situations during the coronavirus disease 2019 (COVID-19) pandemic, the initial enrolment period of 1.5 years was further extended until May 2022 and follow-up completed during a further one year. Participants of all age groups and both sexes, presenting with a minimum of three symptoms corresponding with that derived from proving of AC and those who have not taken any homeopathic medication for the past one week, were included in the study. Those participants who were on medication were kept on a 7-day washout period before enrolment into the study. Participants with a history of uncontrolled or life-threatening disease/systemic illness, any pre-existing psychiatric disorder, under medications such as immuno-suppressants, hormonal therapies, other Ayush therapies or herbal medicines for chronic ailments were excluded from the study. Pregnant or lactating women were also excluded.

Each participant was subjected to a two-level screening procedure before enrolment. A preliminary verbal screening was done by the attending OPD doctor for the presence of symptoms related to the indicated medicine. Potential participants who presented with the relevant symptoms after an initial screening were then attended by the principal investigator (PI) of the respective study site for a detailed second screening. The site PI further confirmed the eligibility of the participant based on the inclusion and exclusion criteria followed by taking the voluntary written consent before enrolment. Written parental consent and verbal assent was obtained from children aged 7 to 12 years, and in those aged 13 to 18 years both consent and assent were recorded in written form.

A detailed case recording was done on the pre-designed case-taking proforma followed by general and systemic examination of the participant. The study materia medica and repertory comprising the proving and literature symptoms of AC amongst the batch of other medicines to be clinically verified were referred by the site PI after the case documentation, to correlate the drug picture of AC with the symptoms of the participant. If the symptoms of the participant were found consistent with any of the proving symptoms recorded in the study materia medica of AC, the participant was prescribed the medicine in either 6C, 30C or 200C potency, prepared as per the German Homoeopathic Pharmacopoeia 11 and dosage based on the need of the case as per the homeopathic principles of repetition and dosage laid out in the *Organon of Medicine* . 20 If the case did not fulfil the symptom similarity criteria, then these participants were considered as an exclusion from the study and treated in the general OPD of the respective homeopathic clinic/hospital.

Follow-up was conducted periodically, based on the improvement in clinical presentation and compliance to visit. As this was an open label study, there were no fixed number of follow-ups for assessment. However, the provision of a maximum of 10 follow-ups was kept in the study. Identical placebo was administered for a definitive period if improvement was observed in the participants after AC, but in cases of status quo the same dosage was repeated once, followed by the next higher potency if change was still not observed. If there was no change in the presenting symptoms of a participant, even after adequate repetition of selected medicine in various potencies, or if there was significant distress in the participant, expressed by the appearance of severe new symptoms, then such participant was referred to be treated in the general OPD. Placebo was also administered if a participant presented with new symptoms of mild character.

### Outcome Assessment

Outcome assessment was based on the response to treatment and changes in MYMOP-2 scores recorded at the baseline and during each subsequent consultation (follow-up visit). MYMOP-2 is a validated, comprehensive, patient-centered and problem-specific outcome measure that enables participants to report their own health outcomes. The usage of the scale in this study helped to verify the therapeutic action of AC by correlating it with the changes observed in Symptom 1 of the scale. The participants were asked to list the two most bothersome symptoms and grade themselves on a 7-point Likert scale ranging from 0 to 6, with 0 being ‘as good as it can be’ and 6 being ‘as bad as it could be’. They were also asked to score the effect of their suffering on their daily activities and general well-being from 0 to 6. Scoring of Symptom 1 and well-being is obligatory, whilst Symptom 2 and activity remain optional. A mean of all the recorded scores is considered as the Profile Score. 17 In the present study, the scores of Symptom 1, activity and well-being were considered for analysis.

Additionally, the participants were asked to rate the changes in their symptoms at each follow-up on the ORIDL scale. ORIDL is a validated 9-point Likert scale with values ranging from −4 (disastrous deterioration) to +4 (cured/ back to normal) that measures participant perception of the outcome of care. Participants, with the help of their doctor, record improvement or deterioration in their originally presenting complaints and its impact on their daily life. A score of +2 or higher was considered as the ‘ORIDL threshold’ because at this level meaningful change in the quality of life is perceived by participants. 18

The study also evaluated the emergence of new clinical symptoms following the administration of the medicine.

### Sample Size

The study was aimed at verifying the therapeutic utility of AC which is a lesser-known remedy in homeopathy; hence, its efficacy in treatment of different clinical conditions was not well defined. Since this was an exploratory study, based on a conservative approach, it was decided that it would be reasonable to assume that 50% of the population would express an improvement in the clinical symptoms after intake of the medicine for different clinical conditions. Assuming a 5% level of significance and 5% error margin, the sample size was determined as follows 21 :



Thus, sample size, *N* = 385, or approximately 400 cases. Considering a potential 20% attrition, the total sample size was increased to 480 cases across 14 sites.

### Statistical Analysis

Data analysis was done following the per-protocol method. Categorical variables were presented as numbers ( *n* ) and percentages (%), whereas the quantitative data were reported as median (interquartile range [IQR]). A *p* -value of <0.05 was considered as statistically significant. The follow-ups in the study were adversely affected by the COVID-19 pandemic situation from 2020 to 2022; hence, data analysis was done for all participants having a minimum of two follow-ups. Out of these data, there were a few participants who had completed 10 follow-ups. A sub-group analysis of these participants (with 10 follow-ups) was done to see whether long-term adherence to the follow-ups resulted in a beneficial change in the treated clinical symptoms.

Analysis was also performed to evaluate the effect of the medicine in various systems of the body, namely musculoskeletal, gastrointestinal, respiratory, etc. To assess the changes in MYMOP-2 from baseline to subsequent visits, the Friedman's test was applied. The Wilcoxon signed-rank test was used to assess changes in ORIDL scores at first and second follow-up as well as at the tenth follow-up. The statistical analysis was performed using GraphPad Prism software v9.0 (San Diego, California, United States).

## Results

**Fig. 1:**
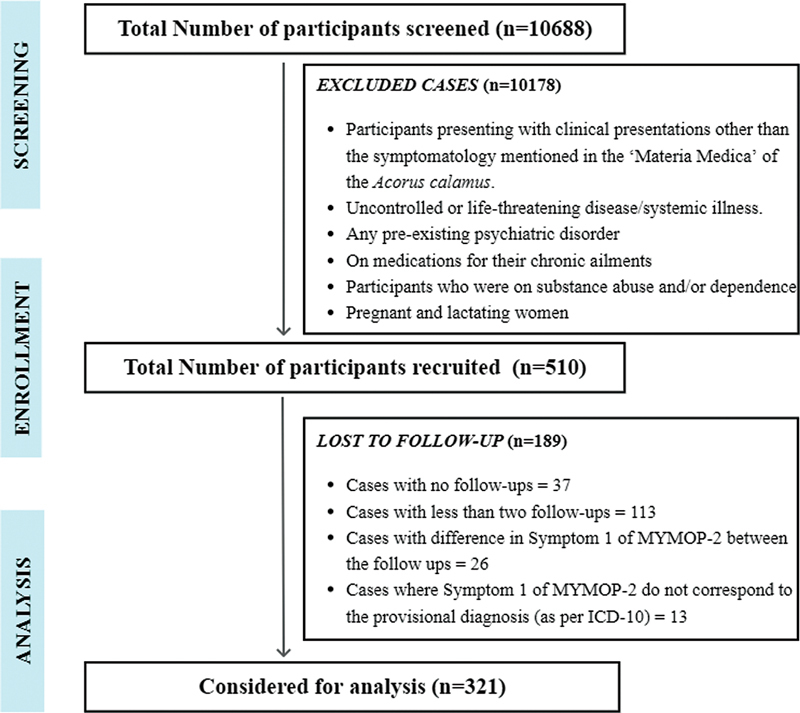
Flow diagram of the study.

Out of the population of 10,688 screened, a total of 510 participants were enrolled prospectively, with 95.23% ( *n* = 10,178) participants excluded at the beginning as they matched the exclusion criteria. A total of 321 participants who completed at least two follow-ups were considered for final analysis. The overall flow of study enrolment is illustrated in Fig. 1 .

### Baseline Demographics

The mean age (±standard deviation) among participants was 37.6 (±14.7). Of the enrolled population, 40.2% (129) were males, and 59.8% (192) were females. The description of the baseline characteristics of the enrolled study population is given in Table 1 .

**Table 1:** Demographic characteristics of the study population

Baseline variable	*n* (%) ( *n* = 321)
Gender	
Male	129 (40.2)
Female	192 (59.8)
Age	
Overall age (mean ± SD, in years)	37.6 ± 14.7
0–10	10 (3.1)
11–20	34 (10.6)
21–30	60 (18.7)
31–40	80 (24.9)
41–50	86 (26.8)
51–60	29 (9)
Above 60	22 (6.9)
Occupation	
Student	50 (15.6)
Homemaker/housewife	136 (42.4)
Employed/self-employed	122 (38)
Retired	9 (2.8)
Health workers/hospital staff	3 (0.9)
Unemployed	1 (0.3)
Religion	
Hindu	275 (85.7)
Christian	23 (7.2)
Muslim	22 (6.9)
Buddhist	1 (0.3)
Marital status	
Married	224 (69.8)
Unmarried	95 (29.6)
Widow	2 (0.6)
General examination (mean ± SD)	
Height (in cm)	156.8 ± 13.1
Weight (in kg)	59.8 ± 13.5
Pulse rate ( *n* = 320)	76.9 ± 7.2
Respiratory rate ( *n* = 319)	18.7 ± 5.8
Systolic blood pressure ( *n* = 312)	118.7 ± 10.5
Diastolic blood pressure ( *n* = 312)	78.1 ± 6.4

Abbreviation: SD, standard deviation.

Note: All variables are expressed as *n* (%).

### Clinically Verified Symptoms

Participants reported a wide range of clinical symptoms as their chief or accompanying complaint, such as pain in different regions of the body (43.6%), headache (34.3%), constipation (30.5%), cough (15.3%), cold/ coryza (12.2%), pain in throat (9.3%), and different types of skin eruptions (7.8%). Further insights into the clinically verified symptoms are provided in Supplementary Table S1 (available in online version only). In this table, the clinically verified symptoms are represented as per anatomical schema in four columns, detailing symptom location, description in repertorial language (from generals to particulars), number of participants who were prescribed the medicine based on the symptom similarity, and the number of participants who showed improvement or were cured after the treatment.

### Clinical Conditions Treated Using *Acorus calamus*

The clinical conditions reported by the 321 participants at baseline were categorised according to International Classification of Diseases version 10 (ICD-10). The majority of participants sought treatment for musculoskeletal, respiratory and digestive symptoms ( Table 2 ). Specifically, the most frequently observed reasons for encounter were myalgia, affecting 26 (8.1%) participants, constipation in 25 (7.8%), arthralgia in 23 (7.2%), cervical spondylosis in 21 (6.5%), headache in 19 (5.9%), and allergic rhinitis in 19 (5.9%) participants ( Table 3 ).

**Table 2:** System-wise most frequently obtained clinical conditions

Classification of clinical conditions with ICD-10 codes	Number of participants
Diseases of the musculoskeletal system and connective tissues (M00–M99)	104
Diseases of the respiratory system (J00–J99)	96
Diseases of the digestive system (K00–K93)	52
Diseases of the skin (L00–L99)	23
Diseases of the eye (H00–H59)	3
Diseases of the ear (H60–H95)	5
Head general symptoms—headache (R50–R69), migraine/cluster headache (G40–G47)	28
General symptoms and signs (R50–R69)—fever	5
Injuries, certain other consequences of external causes (S00–T98)	2
Diseases of the nervous system (G00–G99)	3
Total	321

**Table 3:** Most frequently obtained clinical conditions reported as reason for encounter in OPD

ICD-10 codes	Clinical conditions	Prescribing indications	Number of participants *n* (%)
M79.1	Myalgia	• Pain in upper arm, gradually descends down • Aching pain in legs; agg. motion. Pain gradually disappears at night • Pain in both extremities • Aching pain in both legs • Aching pain in left hand, more in ring finger	26 (8.1%)
K59	Constipation	• Constipation, stool hard, unsatisfactory, had to strain a lot, unfinished sensation	25 (7.8%)
M25.5	Arthralgia/pain in joints	• Pain in right foot in morning on waking up; agg. walking; amel. hard pressure • Dull aching pain in left shoulder; amel. by pressure • Dull, aching pain behind right knee joint	23 (7.2%)
M47.8	Cervical spondylosis	• Pain in back of neck and right shoulder radiates to right hand	21 (6.5%)
R51	Headache	• Heaviness of head; agg. morning. with decreased appetite, no desire to eat, increased thirst for small quantity at short intervals • Aching pain and heaviness in frontal region; agg. loud noise; amel. absolute rest (not moving the head) • Headache, starts from occiput, extends to vertex, settles in both eyes; agg. motion; amel. pressure • Throbbing in frontal region; agg. slight noise; amel. sleep • Dull pain in left side of forehead; agg. least sound. Pain extends to right • Aching pain in right temporal region; amel. pressure. Pain shifted to left • Stitching pain in temporal regions; agg. motion, heat, amel. by hard pressure, cold washing, rest, sleep, lying down • Pulsating and beating pain in both temporal regions; agg. motion, amel. hard pressure • Dull pain in both temporal regions	19 (5.9%)
J 30.9	Allergic rhinitis	• Watery discharge from nose, next day whitish discharge from nose • Coryza, thin, whitish with head congestion and burning in eyes and fever • Blocked nose agg. morning, thick, yellow nasal discharge with heaviness of head • Pricking pain in right side of throat; agg. swallowing, speaking • Pain and tickling in right side of throat with white, thick, ropy phlegm from throat • Cough with thin, white expectoration accompanied with coryza	19 (5.9%)
J 00	Acute nasopharyngitis		17 (5.3%)
J 06.9	URTI		14 (4.4%)
J 32.9	Chronic sinusitis	• Watery discharge from nose, next day whitish discharge from nose • Cough with white expectoration; amel. warm water • Cough with yellow sputum accompanied with stuffed nose • Heaviness of head; agg. morning. • Aching pain and heaviness in frontal region; agg. loud noise; amel. absolute rest (not moving the head)	14 (4.4%)
L20–L30	Dermatitis/eczema	• Vesicular eruptions, itching, burning; amel. rubbing • Eruptions appeared on hand extends to back, stomach, then lower limbs and face. Old eruptions dried up and became black in color	11 (3.4%)
R 05.9	Cough	• Cough with dyspnoea, no expectoration, oppressed feeling in chest • Cough with white expectoration; amel. warm water	11 (3.4%)

Abbreviations: ICD-10, 10th revision of the International Classification of Diseases; URTI, upper respiratory tract infection; agg., aggravated by; amel, ameliorated by.

Notes: 1. Clinical conditions with a minimum of 10 participants reporting have been listed in this table.

2. The table lists common prescribing indications found among most participants. However, it is possible that individual participants may not exhibit all the listed indications.

All variables are expressed as *n* (%).

### Physical and Mental Generals

The physical and mental generals were also observed in the participants as these play a characteristic role in homeopathic prescription. Notably, the participants exhibited distinct physical characteristics, with the most frequent being the following: thermal reaction—chilly (40.5%), increased thirst (43.5%), desire for spicy food (45.8%) and sweets (26.5%), stool – hard (28.6%). Mentally, the most frequently observed traits in the participants included anger (16.9%), irritability (14.3%), quiet disposition (9.4%) and anxiety (9.1%). The details of the physical and mental generals are mentioned in Supplementary Table S2 (available in online version only).

### Clinical Symptoms

The clinical verification process revealed a range of new symptoms that were not previously identified during the drug proving, including sneezing (6.9%), difficulty in breathing (6.5%), flatulence (4.4%) and irritation in the throat (3.7%). These additional clinical symptoms, summarized in Supplementary Table S3 (available in online version only), provide valuable insights and expand our understanding of the therapeutic action of the medicine, highlighting the importance of thorough and comprehensive clinical verification.

### MYMOP-2 and ORIDL at Follow-ups 1 and 2

The complete analysis was done for 321 participants who completed at least two follow-up visits. A sub-group analysis was done for 26 participants who completed the maximum 10 follow-up visits (see below).

A wide range of symptoms reported by participants were measured using MYMOP-2 scale. Most participants scored 3 to 6 on the 6-point MYMOP-2 numerical rating scale for Symptom 1 (98%), activity (96%) and well-being (93%) at baseline. The profile score at baseline ranged between 3 and 6 for 95.9% of the participants. Further baseline data are presented in Table 4 .

**Table 4:** Baseline data of MYMOP -2 scores in participants ( *n* = 321)

Scores	Symptom 1 *n* (%)	Activity *n* (%)	Well-being *n* (%)	Score range	Profile score *n* (%)
0	0 (0.00%)	1 (0.3%)	0 (0.00%)	0– < 1	0 (0.0%)
1	0 (0.00%)	4 (1.2%)	0 (0.00%)	1– < 2	3 (0.9%)
2	5 (1.6%)	8 (2.5%)	21 (6.5%)	2– < 3	10 (3.1%)
3	34 (10.6%)	74 (23.1%)	81 (25.2%)	3– < 4	85 (26.5%)
4	77 (24.0 %)	77 (24.0%)	73 (22.7%)	4– < 5	91 (28.3%)
5	121 (37.7%)	93 (29.0%)	99 (30.8%)	5– < 6	93 (29.0%)
6	84 (26.2%)	64 (19.9%)	47 (14.6%)	6	39 (12.1%)

Note: All variables are expressed as *n* (%).

**Fig. 2:**
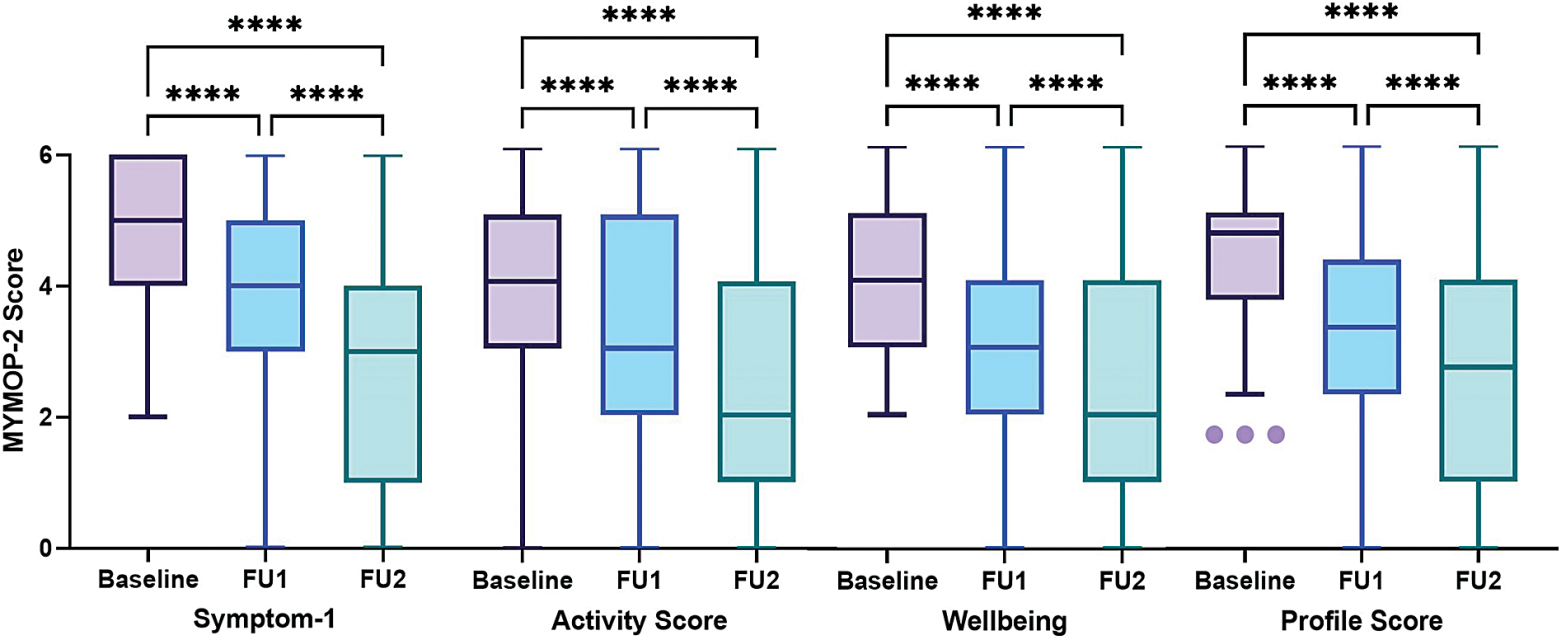
MYMOP-2 scores at baseline, follow-up 1 and follow-up 2. FU, follow-up; MYMOP-2, Measure Yourself Medical Outcome Profile-2.

Each of the MYMOP-2 domain scores at baseline, follow-up 1 and follow-up 2 were compared using Friedman's test and found to be statistically significant ( *p* < 0.0001). The median (IQR) scores of the profile score reduced from 4.7 (3.7–5) at baseline to 2.7 (1–4) at the second follow-up, showing a significant improvement in the overall outcome. Similar significant reductions were observed in the median scores of Symptom 1, activity, and well-being ( Fig. 2 ).

A separate system-wise analysis exploring for the improvement in the symptoms based on the provisional diagnosis of the participant was also performed. Statistically significant improvement ( *p* < 0.0001) was observed in all the domain scores of MYMOP-2 in participants with musculoskeletal complaints ( *n* = 104), respiratory complaints ( *n* = 96), digestive complaints ( *n* = 52), skin ( *n* = 23) and head complaints ( *n* = 28), thereby suggesting the potential action of AC in these categories ( Table 5 ).

**Table 5:** System-wise MYMOP-2 score at baseline, follow-up 1 and follow-up 2

	MYMOP-2	Median (IQR) at baseline	Median (IQR) at follow-up 1	Median (IQR) at follow-up 2	*p* -Value
Musculoskeletal system ( *n* = 104)	Symptom 1	5 (4–5)	4 (3–5)	3 (2–4)	<0.0001
	Activity	4 (4–5)	3.5 (2–5)	3 (1–4)	<0.0001
	Well-being	4 (3–5)	3 (2–4)	2 (1–4)	<0.0001
	Profile score	4.3 (3.7–5)	3.3 (2.4–4.6)	2.7 (1.3–4)	<0.0001
Respiratory complaints ( *n* = 96)	Symptom 1	5 (4–5)	3 (2–4)	2.5 (1–4)	<0.0001
	Activity	4 (3–5)	3 (2–4)	2 (1–4)	<0.0001
	Well-being	4 (3–5)	3 (2–4)	2 (1–4)	<0.0001
	Profile score	4.7 (3.3–5)	3 (2–4)	2.3 (1–3.93)	<0.0001
Digestive system ( *n* = 52)	Symptom 1	5 (4–6)	4 (3–5)	3 (1–5)	<0.0001
	Activity	5 (3.25–6)	4 (3–5)	3 (1–5)	<0.0001
	Well-being	5 (3–5.75)	4 (3–5)	3 (1–5)	<0.0001
	Profile score	5 (3.7–5.7)	3.85 (3–5)	3 (1–5)	<0.0001
Skin ( *n* = 23)	Symptom 1	5 (4–6)	3 (3–4)	2 (1–4)	<0.0001
	Activity	4 (3–5)	3 (2–4)	3 (0–3)	<0.0001
	Well-being	4 (3–5)	3 (2–4)	3 (1–3)	<0.0001
	Profile score	4.7 (3.7–5)	3.7 (2.3–4)	2.7 (1–3.3)	<0.0001
Head complaints ( *n* = 28)	Symptom 1	5 (4–6)	4 (4–5)	3 (2–4)	<0.0001
	Activity	5 (4–5)	4 (3–5)	3 (2–4)	<0.0001
	Well-being	5 (4–5)	4 (3–5)	3 (2–4)	<0.0001
	Profile score	4.85 (4.3–5.3)	4 (3.3–5)	2.85 (1.85–4.23)	<0.0001

Abbreviations: IQR, interquartile range (25 ^th^ percentile–75 ^th^ percentile); MYMOP-2, Measure Yourself Medical Outcome Profile-2.

**Fig. 3:**
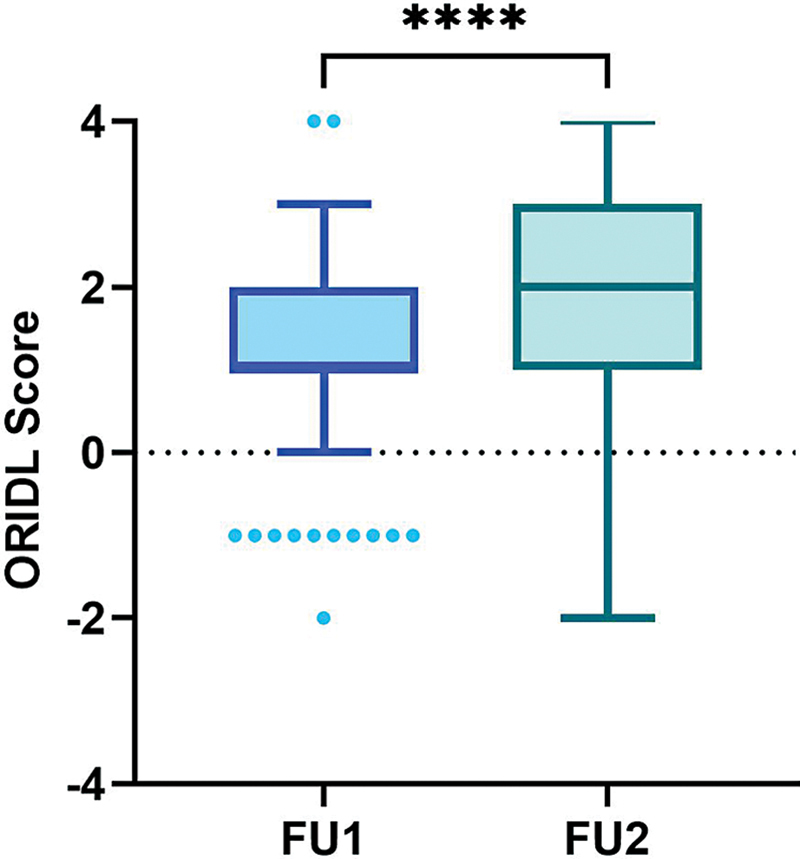
ORIDL scores at follow-up 1 and follow-up 2. FU, follow-up; ORIDL, Outcome in Relation to Impact on Daily Living.

The Wilcoxon signed-rank test was applied to analyze ORIDL scores at follow-ups 1 and 2. The result showed significantly greater improvement at follow-up 2 ( *p* < 0.0001; Fig. 3 ).

### Status of Follow-ups in the Participants

Out of 321 participants, there were 26 who completed the maximum 10 follow-up visits. The remaining 295 participants had different follow-up frequencies: 99 (30.8%) had 2 visits only, 75 (23.4%) had 3 visits, 48 (15%) had 4 visits, 25 (7.8%) had 5 visits, 16 (5%) had 6 visits, 11 (3.4%) had 7 visits, 14 (4.4%) had 8 visits, and 7 (2.2%) had 9 visits.

**Fig. 4:**
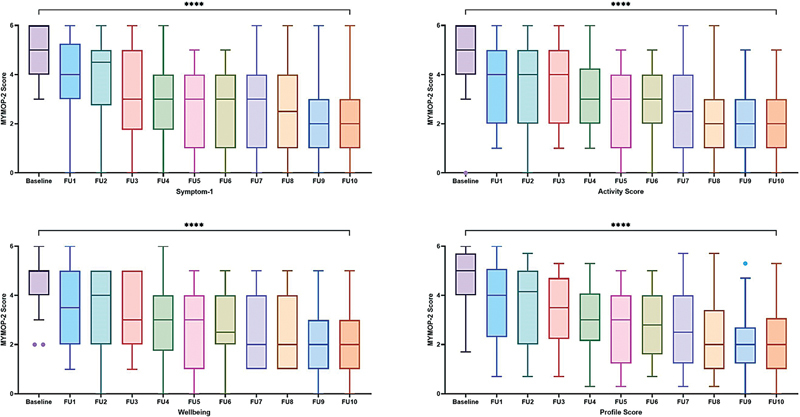
MYMOP-2 scores for participants with 10 follow-ups ( *n* = 26). FU, follow-up; MYMOP-2, Measure Yourself Medical Outcome Profile-2.

Analysis for improvement in 26 participants who completed the study with 10 follow-up visits (V0–V10) was also performed using the Friedman's test. Statistically significant improvement ( *p* < 0.0001) was observed in overall profile score and all the domain scores of MYMOP-2 (including, Symptom 1, activity and well-being; Fig. 4 ).

The ORIDL scores also improved for these 26 participants between follow-up 1 and follow-up 10, with median (IQR) score of 1 (0.75–2) at the 1st follow-up and median 2 (1–3) at the 10th follow-up ( *p* = 0.0028).

## Discussion

Clinical verification is an essential aspect of validating the clinical action of any homeopathic medicine. It involves the confirmation of signs and symptoms recorded during drug proving in patients, thereby substantiating the therapeutic action of the medicine. This study was conducted to verify the symptomatology of AC clinically by determining the change in symptoms after intake of the medicine measured using the validated MYMOP-2 and ORIDL scales.

A total of 510 participants from various locations in India were prescribed AC based on the symptom similarity. However, there was a significant loss to follow-up due to the ongoing COVID-19 pandemic across the country. Hence, the response to the treatment was analyzed on 321 participants who completed at least two follow-ups after the prescription at baseline. A positive response was seen from the first follow-up onwards and it continued up to the last follow-up, as was seen in the MYMOP-2 scores of cases who completed the 10 follow-ups. The analysis of the results highlighted statistically significant and clinically meaningful responses.

The prescription of AC yielded positive responses in participants, with various clinical conditions such as myalgia, constipation, arthralgia/pain in all joints, allergic rhinitis, cervical spondylosis, headache, acute nasopharyngitis, upper respiratory tract infection (URTI), chronic sinusitis, dermatitis/eczema and cough. This observation suggests that it has broad therapeutic effects on various spheres of action including the central nervous system, respiratory, gastrointestinal and musculoskeletal systems, eyes and skin. The study also revealed several physical and mental generals that were not previously observed during the drug proving phase, which may help in expanding the materia medica of the medicine. The study identified statistically significant improvement in the symptoms of the participants, with notable benefits after the second visit in those who completed 10 follow-ups. This suggests that long-term adherence to treatment may lead to enhanced therapeutic effects.

The COVID-19 pandemic and the resulting lockdowns significantly impacted the study, leading to variability in the number of follow-ups among participants. Despite this challenge, an attempt was made to verify the symptomatology of AC through assessment in participants who completed a minimum of two follow-ups. Most participants demonstrated symptom improvement after the first or second follow-up visit.

This research investigated the homeopathic therapeutic perspective of AC for the first time. This medicine was first proved by CCRH and is now clinically verified in a large number of participants from various locations across India. This geographic heterogeneity has served the purpose of including participants from diverse socio-economic, cultural and climatic backgrounds.

Every study has its own limitations. As ours was an open-label design, the study was susceptible to several issues including lack of randomization or blinding, the placebo effect, the regression effect towards the mean, and the risk of various biases associated with selection, performance and confirmation, which might have influenced the study's results. Inclusion based on the symptom similarity of AC may have introduced confirmation bias. Due to the prevailing COVID-19 pandemic, the study suffered a significant risk of random attrition bias, thereby compromising the internal and external validity of the results. The significant loss to follow-ups and fewer enrolments during the study duration led to an extension of the study to achieve the pre-determined sample size. However, the multi-center design with independent site PIs helped to mitigate these biases. The protocol of the study was not pre-published. However, the reporting adhered to the STROBE 19 statement checklist, ensuring comprehensive inclusion of all essential items for observational studies.

The drug pathogenetic trial of AC had been conducted and recorded in a small group of provers and the intensity of symptoms was not ascertained at that time. Hence, all the proving symptoms were given equal importance during clinical verification. Moreover, as the study protocol did not mandate any laboratory-based final diagnosis, we documented the presenting complaints as ‘reason for encounter’. Future studies can be designed to include laboratory confirmation of the final diagnosis.

## Conclusion

The study aimed to verify the symptomatology of AC by ascertaining the change in the symptoms after its administration in individuals with various health conditions. The symptoms previously reported in drug proving were confirmed during the study. Additionally, certain new mental and physical symptoms were documented as clinical symptoms of the medicine. Under the CCRH's Clinical Verification Program, for the first time the validated scales MYMOP-2 and ORIDL were used for the assessment of the results. Statistically significant improvement was observed in participants suffering from various clinical entities such as myalgia, constipation, arthralgia/pain in all joints, allergic rhinitis, cervical spondylosis, headache, acute nasopharyngitis, URTI, chronic sinusitis, dermatitis/eczema and cough. There is a need now for double-blinded or pragmatic randomized controlled trials focusing on the efficacy or effectiveness of AC for the indicated clinical conditions.

## Highlights

- The proving symptoms of the lesser-known homeopathic medicine, *Acorus calamus* , in 6C, 30C and 200C potencies was clinically verified in an open-label study conducted in 14 centers across India.
- A total of 321 participants with a minimum of two follow-ups showed a statistically significant improvement in their symptoms after administration of the medicine, assessed using the MYMOP-2 and ORIDL scales.
- The most frequently presented symptoms which were clinically verified included headache, constipation, cough, coryza, pain in throat, and pain in different regions of the body.
- Several physical and mental generals of *Acorus calamus* were documented for the first time.

**Supplementary Table S1:** Clinically Verified Symptoms of *Acorus calamus*

LOCATION	SYMPTOMS	NUMBER OF PARTICIPANTS PRESCRIBED	NUMBER OF PARTICIPANTS CURED/ IMPROVED
**HEAD**	**Heaviness**	**36**	**34**
	< morning	6	6
	decreased appetite, with	1	1
	increased thirst, with	2	2
	Frontal region	2	2
	**occiput**	**12**	**12**
	drowsiness, with	5	5
	< noise	4	4
	* < morning*	3	3
	* < bending forward*	2	2
	* > pressure*	2	1
	* blocked nose, with*	3	3
	**Pain in head**	**110**	**104**
	> pressure	6	6
	*< noise*	2	2
	*nausea, with*	5	5
	* aching pain with heaviness, right side, radiating right to left*	2	2
	*bursting*	2	2
	*< noise*	2	2
	*dull*	2	2
	*throbbing*	2	2
	*< morning*	2	2
	**frontal region**	**38**	**37**
	left	1	1
	right	3	3
	> pressure	1	1
	< noise	4	4
	> sleep	3	3
	aching	9	9
	< loud noises	1	1
	> rest	1	1
	throbbing	2	2
	> sleep	2	2
	*> rest*	2	2
	*pulsating*	7	7
	*< noise*	6	6
	* > bandaging*	2	2
	* extending to vertex*	2	2
	occipital	6	6
	> lying	1	1
	< sun exposure	1	1
	*> pressure*	4	4
	**temporal**	**37**	**35**
	> pressure	4	4
	aching	8	6
	> pressure	7	5
	*> bandaging*	1	1
	dull	5	5
	*< morning, > pressure, drowsiness, with*	3	3
	**pulsating**	**12**	**12**
	> pressure	7	7
	> *after sleep*	2	2
	*< noise*	7	7
	* hammering, < noise, > after sleep, extending to vertex and occiput*	2	2
			
**EYES**	**Pain**	**19**	**18**
	right	2	2
	> pressure	1	1
	**burning**	**15**	**14**
	redness, with	2	2
	*right*	3	3
	*> pressure*	2	2
**EAR**	Pain	8	7
	right	5	5
	**>** sleep	4	4
	neck, extending to	2	2
	lightning	4	4
			
**NOSE**	**Coryza**	**39**	**39**
	congestion of head, with	2	2
	fever, with	5	5
	*chilliness, with*	2	2
	obstruction, with	6	6
	< morning	5	5
	*< morning*	5	5
	*< change of weather*	3	3
	***<*** *cold things*	3	3
	* < drinking cold water, cold drinks*	7	7
	*< dust*	2	2
	*< ice-cream*	2	2
	*< rainy weather*	2	2
	*< sour things*	2	2
	*> warm drinks*	3	3
	*< winter*	4	4
	***recurrent***	**10**	**10**
	**Discharge**	**72**	**72**
	**thick**	**18**	**18**
	heaviness of head, with	1	1
	thin	9	9
	white	4	4
	**watery**	**34**	**34**
	* < morning, congestion of head, with*	2	2
	** yellow**	**11**	**11**
	heaviness of head, with	1	1
	*morning*	8	8
	*sticky*	3	3
	*yellowish- white*	4	4
	**Obstruction of nose**	**39**	**38**
	**< morning**	**27**	**27**
	*< evening*	2	2
	*< night*	2	2
	* < cold weather, during sleep, while lying down*	3	3
	*< winter*	2	2
	*heaviness of head, with*	7	7
			
**FACE**	Dryness of lips	7	6
	increased thirst, with	2	2
			
**MOUTH**	Bitter taste in the mouth after fever	1	1
	Profuse salivation in mouth	8	8
			
**THROAT**	Inflammation of uvula	3	3
	pain, with	2	2
	**Pain**	**30**	**30**
	ear, extending to	1	1
	pricking	5	5
	right	2	2
	< swallowing	1	1
	*< cold drinks, while coughing*	2	2
	*< sour*	6	6
	*< swallowing*	7	7
	*> warm water gargles*	2	2
	*constricted*	4	4
	Tickling	4	3
			
**STOMACH**	Pain	3	3
	> bending forward	1	1
	*nausea, during*	2	2
	*burning pain in epigastrium*	5	5
	Vomiting	4	4
	with profuse salivation	3	3
			
**ABDOMEN**	**Pain**	**31**	**30**
	frequent stool, with	3	3
	**nausea, with**	**14**	**13**
**RECTUM**	**Constipation**	**98**	**89**
	**unsatisfactory, unfinished, insufficient stool**	**75**	**66**
	voided with strain	3	3
	***ineffectual urging***	12	12
	*< eating, after, in morning*	5	5
	Urging, immediately after meals	1	1
**COUGH**	**Cough**	**49**	**46**
	coryza, with	3	3
	dyspnoea, with	11	10
	chest pain < at night	2	2
	expectoration, with	23	23
	lying down, on	1	1
	**dry**	**17**	**16**
	dyspnoea, with, *> warm water*	2	2
	*< night, > warm water*	3	3
	* > warm drinks, warm saline gargling*	2	2
	*suffocating*	8	7
			
**EXPECTORATION**	Thin	1	1
	**White**	**24**	**24**
	> warm water	2	2
	**Yellow**	**10**	**10**
			
**CHEST**	Oppression when coughing	3	3
	Pain	10	9
	cough, during	3	3
	aching	2	2
	right	1	1
	dull	3	3
	right	2	2
**BACK**	**Pain**	**14**	**14**
	**cervical region**	**35**	**29**
	extending to right hand	7	6
	*drawing*	3	3
	*extending to right hand*	11	6
	*dull aching*	2	2
**EXTREMITIES**	**Eruptions**	**13**	**13**
	black	4	4
	dry	6	6
	upper limb, itching with	3	3
	vesicular	3	3
	hands	2	2
	Pain	8	8
	aching	7	7
	* < motion*	2	2
	**shoulder**	**42**	**42**
	*> pressure*	2	2
	*extending to hand*	4	4
	*right*	2	2
	**left**	**12**	**12**
	aching	8	8
	> pressure	3	3
	*extending to hand*	2	2
	*> pressure*	2	2
	right	8	8
	extending to hand	4	4
	*aching*	4	4
	**knees**	**26**	**23**
	right	6	6
	behind	4	4
	*walking, while*	2	2
	***aching***	**13**	**10**
	*right*	9	7
	*behind*	7	5
	*<motion*	2	2
	**arm**	**10**	**10**
	gradually descends down	8	8
	hands	5	4
	left	3	2
	*aching*	2	2
	thighs	8	8
	right	4	4
	lightening like	3	3
	**legs**	**41**	**38**
	< rest	2	2
	**< walking**	**12**	**11**
	**aching**	**16**	**14**
	< motion	5	4
	*> pressure*	2	2
	*in calf muscles*	5	5
	*right*	2	2
	*> pressure*	4	4
	*left*	3	3
	*< motion*	5	5
	**foot**	**14**	**14**
	right	5	5
	< walking, > pressure	1	1
	*< morning*	4	4
	*walking, while*	3	3
**SLEEP**	**Sleeplessness**	**28**	**25**
	**night**	**27**	**24**
**FEVER**	**Fever**	**15**	**15**
	evening	6	6
	< night	8	8
	bodyache, with	5	5
	thirstlessness, with	3	3
	*coryza, with*	2	2
**SKIN**	**Eruption**	**25**	**24**
	black	7	6
	vesicular	6	6
	burning, with	2	2
	itching, with	3	3
	*burning*	3	3
	*itching*	5	5
	*red*	2	2
	*> rubbing*	3	3
			
**GENERALITIES**	**Pain**	**27**	**27**
	exertion, after	3	3
	> rest	1	1
	sore, bruised	6	6
	*<morning, from exertion*	2	2
	weakness, with	4	4
	*aching*	3	3
	*<morning*	2	2

Note:

Typography used:

1. **Bold:** Symptoms that were found in proving and improved in more than 10 participants.

2. *Italics:* Additional characteristics of clinically verified symptoms, including character, modalities and associated/ concomitant symptoms that emerged during the study but were not observed during drug proving of AC.

**Supplementary Table S2:** Physical and Mental Generals of the participants at baseline

Physical generals ( *n* = 321)		n (%)
**Thermal reactions**	
Chilly	130 (40.5)
Hot	108 (33.6)
Ambithermal	83 (25.9)
**Desire**	
Spicy	147(45.8)
Sweets	85(26.5)
Salt/Salty food	39(12.1)
Fish	33(10.3)
Sour	31(9.7)
**Aversion**	
Sweets	26(8.1)
Sour	13(4.0)
**Intolerance to**	
Cold food/drink	12(3.7)
**Appetite**	
Normal	162(50.5)
Increased	85(26.5)
Decreased	61(19.0)
Loss of appetite	13(4.0)
**Thirst**	
Increased	127 (39.6)
Decreased	61 (19.0)
**Stool**	
Constipated/ Unsatisfactory/insufficient/incomplete	98 (30.5)
Clear/Satisfactory/ normal	93 (29.0)
Regular	82 (25.5)
Hard	86 (26.8)
Semi-solid	24 (7.5)
**Urine**	
Regular/satisfactory	186 (57.9)
Clear	72 (22.4)
Straw colored	30 (9.3)
Frequent	12 (3.7)
**Tongue**	
Moist tongue	170 (53.0)
Clean tongue	182 (56.7)
Dry tongue	47 (14.6)
White coated	51 (15.9)
Normal	19 (5.9)
White coated in middle	12 (3.7)
**Taste**	
Normal	289 (90.0)
**Perspiration**	
Normal	133 (41.4)
Profuse/excessive	90 (28.0)
Scanty	51 (15.9)
Generalised	21 (5.9)
Offensive	11 (3.1)
**Sleep**	
Normal/sound/good/satisfactory/ refreshing	199 (61.4)
Disturbed	47 (13.7)
Reduced/ less sleep/lack of	36 (11.2)
Deep	31 (9.7)
**Mind symptoms (** ***n*** ** = 321)**	
Anger/easily angered/anger at trifle	54 (16.8)
Irritable	45 (14)
Anxiety/anxious/ mental tension	26 (8.1)
Calm/quiet/silent	31 (9.7)
Fear	15 (4.7)
Weeping/tearful	24 (7.5)
Desire company	17 (5.3)
Mild/gentle	19 (5.9)
Cooperative	12 (3.7)
Forgetful	15 (4.7)
Consolation aggravation	12 (3.7)
Emotional/sensitive	14 (4.4)

Abbreviation: n, number of participants.

Notes: Physical and mental generals observed/verified in a minimum of ten participants have been listed in this table.

All variables are expressed as n (%).

**Supplementary Table S3:** Clinical Symptoms of *Acorus calamus*

LOCATION	SYMPTOMS	NUMBER OF PARTICIPANTS PRESCRIBED	NUMBER OF PARTICIPANTS CURED/ IMPROVED
**HEAD**	Pain, vertex	4	4
	Vertigo	5	4
**EYES**	Heaviness	3	2
	Lachrymation	5	5
	acrid, coryza, with	2	2
**NOSE**	Postnasal Dripping	8	8
	thick	6	6
	white	2	2
	yellow	5	5
	Smell, diminished	2	2
	**Sneezing**	**22**	**20**
	< morning	5	5
	< dust	4	4
	< pollens	2	2
	coryza, with	5	4
	excessive	3	3
**MOUTH**	Aphthae	2	2
	Dryness	3	2
	tongue	5	5
**THROAT**	Dryness	4	4
	**Irritation**	**12**	**12**
	< pollens	2	2
	Tingling in throat	2	2
	thick sputum, with	1	1
**STOMACH**	Sour eructations	3	3
**ABDOMEN**	**Distension of abdomen**	**10**	**10**
	< spicy food, after	2	2
	> by passing flatus	3	3
	**Flatulence**	**14**	**14**
	Fullness of abdomen	3	3
	flatulence, with	2	2
	Heaviness of abdomen	3	3
	Pain in lower abdomen	2	2
**RECTUM**	Bleeding, stool, during	7	7
	Diarrhea, pain in abdomen, with	5	5
	Pain, tenesmus	9	9
	stool, during	2	2
**GENITALIA FEMALE**	Leucorrhoea	2	2
	Menses, clotted	3	2
	Pain during menses	5	4
**LARYNX AND TRACHEA**	Hoarseness of voice	2	2
	Tickling	4	4
**RESPIRATION**	**Difficult**	**21**	**20**
**EXPECTORATION**	night	3	3
	< lying down	2	2
	Scanty	2	2
	Thick	8	8
**CHEST**	Heaviness in chest	3	2
**BACK**	Pain, dorsal region	3	3
	> pressure	3	3
	between scapulae	3	3
	nape of neck	7	7
	< bending forward, extending to upper limbs, with vertigo	3	3
	extending to shoulder	4	4
	**lumbar region**	**10**	**10**
	< morning	3	3
	Stiffness in nape of neck	2	2
**EXTREMITIES**	Coldness, lower limb, in	3	3
	Pain, elbow	3	3
	left	2	2
	finger, joints	2	2
	upper limb	6	6
	forearm	3	3
	> pressure	2	2
	third finger, aching	2	2
	lower limbs	5	5
	< motion	3	3
	heels	3	3
	Tingling	2	2
**SKIN**	Itching	3	3
**GENERALITIES**	Weakness	5	5

Note:

Typography used:

1. **Bold:** Symptoms that were found in and improved in more than 10 participants.
