# Supplementary material for: Exploring the Homeopathic Therapeutic Potential of Acorus calamus : A Multi-center Open-Label Clinical Verification Study
Source: Homeopathy. 2025 Sep 8;115(2):78–88. doi: 10.1055/a-2625-0202 (PMC13120845; doi:10.1055/a-2625-0202)
Supplement: Supplementary file 1 — Supplementary Material [file 10-1055-a-2625-0202-s2500020.pdf]

Supplementary Table S1 Clinically Verified Symptoms of *Acorus calamus*

LOCATION	SYMPTOMS	NUMBER OF PARTICIPANTS PRESCRIBED	NUMBER OF PARTICIPANTS CURED/ IMPROVED
HEAD	**Heaviness**	**36**	**34**
	< morning	6	6
	decreased appetite, with	1	1
	increased thirst, with	2	2
	Frontal region	2	2
	**occiput**	**12**	**12**
	drowsiness, with	5	5
	< noise	4	4
	< morning	3	3
	< bending forward	2	2
	> pressure	2	1
	blocked nose, with	3	3
	**Pain in head**	**110**	**104**
	> pressure	6	6
	< noise	2	2
	nausea, with	5	5
	aching pain with heaviness, right side, radiating right to left	2	2
	bursting	2	2
	< noise	2	2
	dull	2	2
	throbbing	2	2
	< morning	2	2
	**frontal region**	**38**	**37**
	left	1	1
	right	3	3
	> pressure	1	1
	< noise	4	4
	> sleep	3	3
	aching	9	9
	< loud noises	1	1
	> rest	1	1
	throbbing	2	2
	> sleep	2	2
	> rest	2	2
	pulsating	7	7
	< noise	6	6
	> bandaging	2	2
	extending to vertex	2	2
	occipital	6	6
	> lying	1	1
	< sun exposure	1	1
	> pressure	4	4

 © 2025. The Author(s).

Supplementary Table S1 (*Continued*)

LOCATION	SYMPTOMS	NUMBER OF PARTICIPANTS PRESCRIBED	NUMBER OF PARTICIPANTS CURED/ IMPROVED
	**temporal**	**37**	**35**
	> pressure	4	4
	aching	8	6
	> pressure	7	5
	> *bandaging*	1	1
	dull	5	5
	< *morning,* > *pressure, drowsiness, with*	3	3
	**pulsating**	**12**	**12**
	> pressure	7	7
	> *after sleep*	2	2
	< *noise*	7	7
	hammering, < *noise,* > *after sleep, extending to vertex and occiput*	2	2
EYES	**Pain**	**19**	**18**
	right	2	2
	> pressure	1	1
	**burning**	**15**	**14**
	redness, with	2	2
	right	3	3
	> *pressure*	2	2
EAR	Pain	8	7
	right	5	5
	> sleep	4	4
	neck, extending to	2	2
	lightning	4	4
NOSE	**Coryza**	**39**	**39**
	congestion of head, with	2	2
	fever, with	5	5
	chilliness, with	2	2
	obstruction, with	6	6
	< morning	5	5
	< *morning*	5	5
	< *change of weather*	3	3
	< *cold things*	3	3
	< *drinking cold water, cold drinks*	7	7
	< *dust*	2	2
	< *ice-cream*	2	2
	< *rainy weather*	2	2
	< *sour things*	2	2
	> *warm drinks*	3	3
	< *winter*	4	4

(*Continued*)

 © 2025. The Author(s).

Supplementary Table S1 (*Continued*)

LOCATION	SYMPTOMS	NUMBER OF PARTICIPANTS PRESCRIBED	NUMBER OF PARTICIPANTS CURED/ IMPROVED
	recurrent	**10**	**10**
	**Discharge**	**72**	**72**
	**thick**	**18**	**18**
	heaviness of head, with	1	1
	thin	9	9
	white	4	4
	**watery**	**34**	**34**
	< *morning, congestion of head, with*	2	2
	**yellow**	**11**	**11**
	heaviness of head, with	1	1
	morning	8	8
	sticky	3	3
	yellowish- white	4	4
	**Obstruction of nose**	**39**	**38**
	< **morning**	**27**	**27**
	< *evening*	2	2
	< *night*	2	2
	< *cold weather, during sleep, while lying down*	3	3
	< *winter*	2	2
	heaviness of head, with	7	7
FACE	Dryness of lips	7	6
	increased thirst, with	2	2
MOUTH	Bitter taste in the mouth after fever	1	1
	Profuse salivation in mouth	8	8
THROAT	Inflammation of uvula	3	3
	pain, with	2	2
	**Pain**	**30**	**30**
	ear, extending to	1	1
	pricking	5	5
	right	2	2
	< swallowing	1	1
	< *cold drinks, while coughing*	2	2
	< *sour*	6	6
	< *swallowing*	7	7
	> *warm water gargles*	2	2
	constricted	4	4
	Tickling	4	3
STOMACH	Pain	3	3
	> bending forward	1	1

 © 2025. The Author(s).

Supplementary Table S1 (*Continued*)

LOCATION	SYMPTOMS	NUMBER OF PARTICIPANTS PRESCRIBED	NUMBER OF PARTICIPANTS CURED/ IMPROVED
	nausea, during	2	2
	burning pain in epigastrium	5	5
	Vomiting	4	4
	with profuse salivation	3	3
ABDOMEN	**Pain**	**31**	**30**
	frequent stool, with	3	3
	**nausea, with**	**14**	**13**
RECTUM	**Constipation**	**98**	**89**
	**unsatisfactory, unfinished, insufficient stool**	**75**	**66**
	voided with strain	3	3
	ineffectual urging	12	12
	< eating, after, in morning	5	5
	Urging, immediately after meals	1	1
COUGH	**Cough**	**49**	**46**
	coryza, with	3	3
	dyspnoea, with	11	10
	chest pain < at night	2	2
	expectoration, with	23	23
	lying down, on	1	1
	**dry**	**17**	**16**
	dyspnoea, with, > *warm water*	2	2
	< night, > warm water	3	3
	> warm drinks, warm saline gargling	2	2
	suffocating	8	7
EXPECTORATION	Thin	1	1
	**White**	**24**	**24**
	> warm water	2	2
	**Yellow**	**10**	**10**
CHEST	Oppression when coughing	3	3
	Pain	10	9
	cough, during	3	3
	aching	2	2
	right	1	1
	dull	3	3
	right	2	2
BACK	**Pain**	**14**	**14**
	**cervical region**	**35**	**29**
	extending to right hand	7	6
	drawing	3	3
	extending to right hand	11	6

(*Continued*)

 © 2025. The Author(s).

Supplementary Table S1 (*Continued*)

LOCATION	SYMPTOMS	NUMBER OF PARTICIPANTS PRESCRIBED	NUMBER OF PARTICIPANTS CURED/ IMPROVED
	dull aching	2	2
EXTREMITIES	**Eruptions**	**13**	**13**
	black	4	4
	dry	6	6
	upper limb, itching with	3	3
	vesicular	3	3
	hands	2	2
	Pain	8	8
	aching	7	7
	< motion	2	2
	**shoulder**	**42**	**42**
	> pressure	2	2
	extending to hand	4	4
	right	2	2
	**left**	**12**	**12**
	aching	8	8
	> pressure	3	3
	extending to hand	2	2
	> pressure	2	2
	right	8	8
	extending to hand	4	4
	aching	4	4
	**knees**	**26**	**23**
	right	6	6
	behind	4	4
	walking, while	2	2
	aching	**13**	**10**
	right	9	7
	behind	7	5
	<motion	2	2
	**arm**	**10**	**10**
	gradually descends down	8	8
	hands	5	4
	left	3	2
	aching	2	2
	thighs	8	8
	right	4	4
	lightening like	3	3
	**legs**	**41**	**38**
	< rest	2	2
	< **walking**	**12**	**11**
	**aching**	**16**	**14**
	< motion	5	4

 © 2025. The Author(s).

Supplementary Table S1 (*Continued*)

LOCATION	SYMPTOMS	NUMBER OF PARTICIPANTS PRESCRIBED	NUMBER OF PARTICIPANTS CURED/ IMPROVED
	> pressure	2	2
	in calf muscles	5	5
	right	2	2
	> pressure	4	4
	left	3	3
	< motion	5	5
	**foot**	**14**	**14**
	right	5	5
	< walking, > pressure	1	1
	< morning	4	4
	walking, while	3	3
SLEEP	**Sleeplessness**	**28**	**25**
	**night**	**27**	**24**
FEVER	**Fever**	**15**	**15**
	evening	6	6
	< night	8	8
	bodyache, with	5	5
	thirstlessness, with	3	3
	coryza, with	2	2
SKIN	**Eruption**	**25**	**24**
	black	7	6
	vesicular	6	6
	burning, with	2	2
	itching, with	3	3
	burning	3	3
	itching	5	5
	red	2	2
	> rubbing	3	3
GENERALITIES	**Pain**	**27**	**27**
	exertion, after	3	3
	> rest	1	1
	sore, bruised	6	6
	<morning, from exertion	2	2
	weakness, with	4	4
	aching	3	3
	<morning	2	2

Note:
Typography used:
1. **Bold:** Symptoms that were found in proving and improved in more than 10 participants.
2. *Italics:* Additional characteristics of clinically verified symptoms, including character, modalities and associated/ concomitant symptoms that emerged during the study but were not observed during drug proving of AC.

Supplementary Table S2 Physical and Mental Generals of the participants at baseline

Physical generals ($n = 321$)	n (%)
**Thermal reactions**	
Chilly	130 (40.5)
Hot	108 (33.6)
Ambithermal	83 (25.9)
**Desire**	
Spicy	147(45.8)
Sweets	85(26.5)
Salt/Salty food	39(12.1)
Fish	33(10.3)
Sour	31(9.7)
**Aversion**	
Sweets	26(8.1)
Sour	13(4.0)
**Intolerance to**	
Cold food/drink	12(3.7)
**Appetite**	
Normal	162(50.5)
Increased	85(26.5)
Decreased	61(19.0)
Loss of appetite	13(4.0)
**Thirst**	
Increased	127 (39.6)
Decreased	61 (19.0)
**Stool**	
Constipated/ Unsatisfactory/ insufficient/incomplete	98 (30.5)
Clear/Satisfactory/ normal	93 (29.0)
Regular	82 (25.5)
Hard	86 (26.8)
Semi-solid	24 (7.5)
**Urine**	
Regular/satisfactory	186 (57.9)
Clear	72 (22.4)
Straw colored	30 (9.3)
Frequent	12 (3.7)
**Tongue**	
Moist tongue	170 (53.0)
Clean tongue	182 (56.7)
Dry tongue	47 (14.6)
White coated	51 (15.9)
Normal	19 (5.9)
White coated in middle	12 (3.7)
**Taste**	
Normal	289 (90.0)
**Perspiration**	
Normal	133 (41.4)
Profuse/excessive	90 (28.0)
Scanty	51 (15.9)
Generalised	21 (5.9)
Offensive	11 (3.1)
**Sleep**	
Normal/sound/good/satisfactory/ refreshing	199 (61.4)
Disturbed	47 (13.7)
Reduced/ less sleep/lack of	36 (11.2)
Deep	31 (9.7)
**Mind symptoms ($n = 321$)**	
Anger/easily angered/anger at trifle	54 (16.8)
Irritable	45 (14)
Anxiety/anxious/ mental tension	26 (8.1)
Calm/quiet/silent	31 (9.7)
Fear	15 (4.7)
Weeping/tearful	24 (7.5)
Desire company	17 (5.3)
Mild/gentle	19 (5.9)
Cooperative	12 (3.7)
Forgetful	15 (4.7)
Consolation aggravation	12 (3.7)
Emotional/sensitive	14 (4.4)

Abbreviation: n, number of participants.
Notes: Physical and mental generals observed/verified in a minimum of ten participants have been listed in this table.
All variables are expressed as n (%).

 © 2025. The Author(s).

Supplementary Table S3 Clinical Symptoms of *Acorus calamus*

LOCATION	SYMPTOMS	NUMBER OF PARTICIPANTS PRESCRIBED	NUMBER OF PARTICIPANTS CURED/ IMPROVED
HEAD	Pain, vertex	4	4
	Vertigo	5	4
EYES	Heaviness	3	2
	Lachrymation	5	5
	acrid, coryza, with	2	2
NOSE	Postnasal Dripping	8	8
	thick	6	6
	white	2	2
	yellow	5	5
	Smell, diminished	2	2
	**Sneezing**	**22**	**20**
	< morning	5	5
	< dust	4	4
	< pollens	2	2
	coryza, with	5	4
	excessive	3	3
MOUTH	Aphthae	2	2
	Dryness	3	2
	tongue	5	5
THROAT	Dryness	4	4
	**Irritation**	**12**	**12**
	< pollens	2	2
	Tingling in throat	2	2
	thick sputum, with	1	1
STOMACH	Sour eructations	3	3
ABDOMEN	**Distension of abdomen**	**10**	**10**
	< spicy food, after	2	2
	> by passing flatus	3	3
	**Flatulence**	**14**	**14**
	Fullness of abdomen	3	3
	flatulence, with	2	2
	Heaviness of abdomen	3	3
	Pain in lower abdomen	2	2
RECTUM	Bleeding, stool, during	7	7
	Diarrhea, pain in abdomen, with	5	5
	Pain, tenesmus	9	9
	stool, during	2	2
GENITALIA FEMALE	Leucorrhoea	2	2
	Menses, clotted	3	2
	Pain during menses	5	4
LARYNX AND TRACHEA	Hoarseness of voice	2	2
	Tickling	4	4

(*Continued*)

 © 2025. The Author(s).

Supplementary Table S3 (*Continued*)

LOCATION	SYMPTOMS	NUMBER OF PARTICIPANTS PRESCRIBED	NUMBER OF PARTICIPANTS CURED/ IMPROVED
RESPIRATION	**Difficult**	**21**	**20**
EXPECTORATION	night	3	3
	< lying down	2	2
	Scanty	2	2
	Thick	8	8
CHEST	Heaviness in chest	3	2
BACK	Pain, dorsal region	3	3
	> pressure	3	3
	between scapulae	3	3
	nape of neck	7	7
	< bending forward, extending to upper limbs, with vertigo	3	3
	extending to shoulder	4	4
	**lumbar region**	**10**	**10**
	< morning	3	3
	Stiffness in nape of neck	2	2
EXTREMITIES	Coldness, lower limb, in	3	3
	Pain, elbow	3	3
	left	2	2
	finger, joints	2	2
	upper limb	6	6
	forearm	3	3
	> pressure	2	2
	third finger, aching	2	2
	lower limbs	5	5
	< motion	3	3
	heels	3	3
	Tingling	2	2
SKIN	Itching	3	3
GENERALITIES	Weakness	5	5

Note:
Typography used:
1. **Bold:** Symptoms that were found in and improved in more than 10 participants.

 © 2025. The Author(s).
